# Enhancing astronaut performance using sensorimotor adaptability training

**DOI:** 10.3389/fnsys.2015.00129

**Published:** 2015-09-16

**Authors:** Jacob J. Bloomberg, Brian T. Peters, Helen S. Cohen, Ajitkumar P. Mulavara

**Affiliations:** ^1^Neuroscience Laboratories, Biomedical Research and Environmental Sciences Division, NASA/Johnson Space CenterHouston, TX, USA; ^2^Wyle Science, Technology, and Engineering GroupHouston, TX, USA; ^3^Bobby R. Alford Department of Otolaryngology Head and Neck Surgery, Baylor College of MedicineHouston, TX, USA; ^4^Universities Space Research AssociationHouston, TX, USA

**Keywords:** spaceflight, countermeasures, training, motor learning, plasticity

## Abstract

Astronauts experience disturbances in balance and gait function when they return to Earth. The highly plastic human brain enables individuals to modify their behavior to match the prevailing environment. Subjects participating in specially designed variable sensory challenge training programs can enhance their ability to rapidly adapt to novel sensory situations. This is useful in our application because we aim to train astronauts to rapidly formulate effective strategies to cope with the balance and locomotor challenges associated with new gravitational environments—enhancing their ability to “learn to learn.” We do this by coupling various combinations of sensorimotor challenges with treadmill walking. A unique training system has been developed that is comprised of a treadmill mounted on a motion base to produce movement of the support surface during walking. This system provides challenges to gait stability. Additional sensory variation and challenge are imposed with a virtual visual scene that presents subjects with various combinations of discordant visual information during treadmill walking. This experience allows them to practice resolving challenging and conflicting novel sensory information to improve their ability to adapt rapidly. Information obtained from this work will inform the design of the next generation of sensorimotor countermeasures for astronauts.

## Introduction

Spaceflight induces changes in multiple physiological systems including muscle atrophy, cardiovascular deconditioning and disruption in sensorimotor function. These changes can impact the ability of astronauts to perform mission critical tasks. Microgravity exposure results in an adaptive central reinterpretation of information from multiple sensory sources to produce a sensorimotor state appropriate for motor actions in this unique environment (Paloski et al., 1992, 1994; Reschke et al., 1994), but this new adaptive state is no longer appropriate for the 1G gravitational environment on Earth. Therefore, upon return, a reorganization in sensorimotor state is required that is appropriate to 1G. During these transitions, astronauts experience deficits in both perceptual and motor functions (Kozlovskaya et al., 1981; Reschke et al., 1994, 1998; Clement and Reschke, 2008). Post-flight locomotor control and segmental coordination show changes that include disruption in spatial orientation during overground walking (Glasauer et al., 1995), alterations in muscle activation variability (Layne et al., 1997, 1998, 2001, 2004), modified lower limb kinematics (McDonald et al., 1996; Courtine et al., 2002; Bloomberg and Mulavara, 2003; Miller et al., 2010), alterations in head-trunk coordination (Bloomberg et al., 1997; Bloomberg and Mulavara, 2003; Mulavara et al., 2012), reduced visual acuity during walking (Peters et al., 2011), and alteration in the selection of appropriate landing strategies after jumping (Newman et al., 1997; Courtine and Pozzo, 2004). Astronauts also show impaired postflight functional mobility in terms of their ability to complete an obstacle course (Mulavara et al., 2010). In this study astronauts tested 1 day after landing following 6 months in space increased their time to complete the course by 48% compared to their pre-flight times. The average time to recover to within 95% of their pre-flight times was 15 days. Similar recovery curves for changes in postural stability control measured using posturography with dynamic head movements have been observed after 6 month spaceflights (Wood et al., 2011). These postflight changes in postural and locomotor control might have adverse consequences if a rapid egress were required following a long-duration mission or a Mars landing. Early Mars mission objectives might be compromised by significant postflight postural and gait dysfunction. These changes have implications for potential emergency egress scenarios particularly where support personnel will not be available to aid crewmembers. Orion and other commercial vehicles are currently designed for a parachuted landing on water after long-duration exploration class missions. For safety and operational reasons, returning crewmembers might need to egress the vehicle within a few minutes after a water landing under various sea state conditions. In such water landing scenarios, the interaction between the adapted microgravity state and the prevailing unstable support surface might increase the risk associated with an emergency egress situation. Currently, no operational countermeasure is targeted to mitigate postflight gait dysfunction.

**Figure 1:**
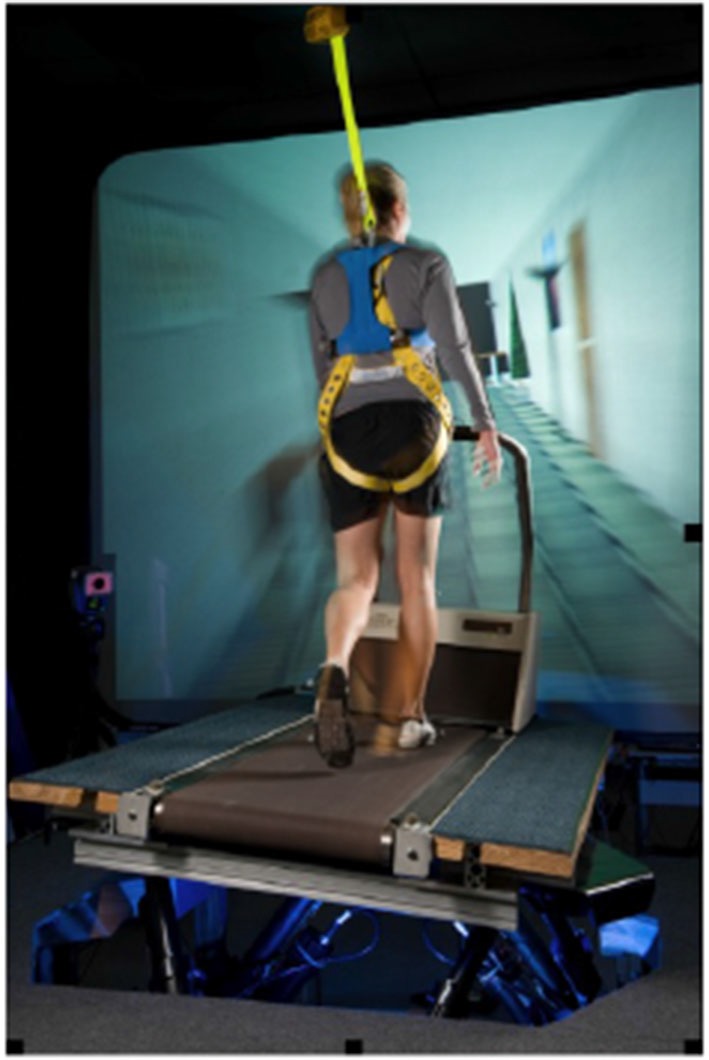
**The Sensorimotor Adaptability (SA) Training System is comprised of a treadmill mounted on a motion base platform.** The support surface is manipulated during walking to challenge a subject’s balance and gait stability. Additional sensory variation is presented with a visual scene that is programmed to conflict with the motion of the support surface. Subjects solve different combinations of support surface movement and visual scene motion, improving their ability to adapt while experiencing challenging and conflicting sensory environments.

Over the last several years with these issues in mind we have developed a training program to enhance the ability to adapt to novel sensory environments: Sensorimotor Adaptability (SA) training. The premise is that by teaching individuals to solve a *class* of sensorimotor, balance, and/or locomotor challenges, rather than a specific, isolated scenario, they will develop more robust adaptation techniques and will be able to select appropriate strategies faster, especially in situations where they might encounter completely unrehearsed and untrained perturbations to their balance and gait control. Although no laboratory setting or set of exercises can perfectly prepare astronauts for how their first steps on the Martian surface will feel after a 6 month journey through space, we expect that SA training will expedite their successful transition to a new sensory environment in much the same way it did in our ground-based studies. To perform SA training subjects walk on a treadmill that is mounted on a six degree-of-freedom motion base in front of a large screen used to provide visual stimuli, so that both the support surface and the visual input can be manipulated simultaneously (See Figure 1). The studies described below summarize our previous research to validate the concept of adaptive generalization as a training technique and to optimize its delivery to subjects in our treadmill and motion base system.

## Exposure to Task Variability Leads to Enhanced Generalization of Motor Skills

The motor learning literature describes two ways to organize a practice session: practice is either blocked, where one task is practiced repeatedly, or variable, in which several task variations are performed. Schmidt’s schema theory of motor learning suggested that the motor system stores generalized motor programs for classes of movement problems (Schmidt, 1975). The performer stores information about initial conditions for each trial, specifics surrounding the motor response that occurred, the movement’s sensory consequences, and the final outcome. This set of relationships makes up the schema. Motor learning occurs over trials because, based on feedback, subsequent performance is adjusted. Thus, novel tasks can be learned if they fall within a class of movement problems already solved by the performer.

This theory suggests that by varying the conditions of practice, critical features of the task are retained while the motor schema is continually refined. This theory also postulates that improved generalization results from variable practice. Variable practice sessions allow the subject to explore options and solutions to achieve the goal of the task they are given. When presented with a novel task, the subject trained with varied practice is more efficient at recalling appropriate movement parameters to successfully and efficiently complete the task (McCracken and Stelmach, 1977; Shea and Morgan, 1979; Catalano and Kleiner, 1984; Sekiya et al., 1994; Sherwood, 1996). Importantly, variable practice approaches have been used in applied contexts to improve motor skills required for a number of different activities including volleyball (Bortoli et al., 1992), racket sports (Wrisberg and Liu, 1991; Green et al., 1995), basketball (Landin et al., 1993; Shoenfelt et al., 2002), soccer (Anderson and Sidaway, 1994; Li and Lima, 2002), throwing (Kerr and Booth, 1978), and catching (Bennett et al., 1999). Variable practice training has also been proposed as a method to improve the motor control of children with Down syndrome (Latash et al., 2002) and in rehabilitation regimens (Krakauer, 2006).

Variable practice exposes the subject to task variation and enables the subject to rehearse the skill using several broader modifications of the movement. Variable practice allows the subject to explore different movement parameters to successfully complete the given task. Over time, the individual is better able to adapt to and successfully navigate through the given environment. Variable practice may increase retention because it requires additional processing during skill acquisition, which facilitates retention of that skill. Rather than simply recalling the previous trial, changing tasks from trial to trial forces the subject to generate a new “solution” each time the task is performed (Schmidt and Lee, 2005). Variable practice encourages subjects to generalize motor learning to many novel variations they might face in the future.

## Enhancing Sensorimotor Adaptability Through Training

A training program that includes both task variability and repeated exposure to sensorimotor challenges can result in faster adaptation to novel sensory environments. The capacity to “learn to learn” or to enhance SA was first confirmed by Welch and colleagues (Welch et al., 1993) who exposed subjects to prismatic displacement of the visual scene and showed that subjects who repeatedly adapted and readapted to prismatic displacement developed the ability to adapt faster to novel visual displacements. They described this increased capacity for adaptability as *adaptive generalization*. Adaptive generalization of motor skills can be enhanced through training including both manual control (Welch et al., 1993; Shadmehr and Moussavi, 2000; Bock et al., 2001; Roller et al., 2001; Seidler, 2004; Stroud et al., 2005) and locomotion (van Hedel et al., 2002; Lam and Dietz, 2004; Cohen et al., 2005; Mulavara et al., 2009; Batson et al., 2011). This type of training is effective in rehabilitating patients with balance control problems (Pavlou et al., 2004; Silsupadol et al., 2006; Suárez et al., 2006), gait disturbances (Baram and Miller, 2006; Fung et al., 2006) and manual control and perceptual-motor disturbances (Adamovich et al., 2004; Rizzo et al., 2004; Holden, 2005; Krakauer, 2006). These studies all support the notion that performers who practice solving a class of motor problems improve their ability to adapt or “learn to learn”. Hence, they may learn to generalize better than performers who practice generating only one solution. Other work has shown adaptive generalization across motor responses. For example, subjects exposed to the visual distortion of prism adaptation during walking generalize this adaptation to reaching, (Morton and Bastian, 2004). Additionally, adaptation to a visual rotation can transfer between movement categories; from a pointing task to a tracking task and* vice versa* (Abeele and Bock, 2003). Introducing movement variability through training may serve to optimize the motor learning system by introducing some degree of chaotic structure that enhances adaptability (Stergiou and Decker, 2011). Supplemental virtual reality exposure during rehabilitation can optimize variability and increase motor function adaptability in a variety of rehabilitation settings and with a diverse set of clinical problems (Rose et al., 1996; Wilson et al., 1997; Myers and Laenger, 1998; Tarr and Warren, 2002; Pavlou et al., 2004; Holden, 2005; Fung et al., 2006; Krakauer, 2006; Whitney et al., 2006; Oddsson et al., 2007; Adamovich et al., 2009; Moreira et al., 2013).

These studies support the concept that a training program that exposes astronauts to variations in sensory input and to balance challenges with repeated adaptive transitions among states will enhance the ability to learn how to assemble and reassemble appropriate motor patterns in novel sensory environments like that encountered after landing on Mars.

## Developing a Sensorimotor Adaptability Training Program

**Figure 2:**
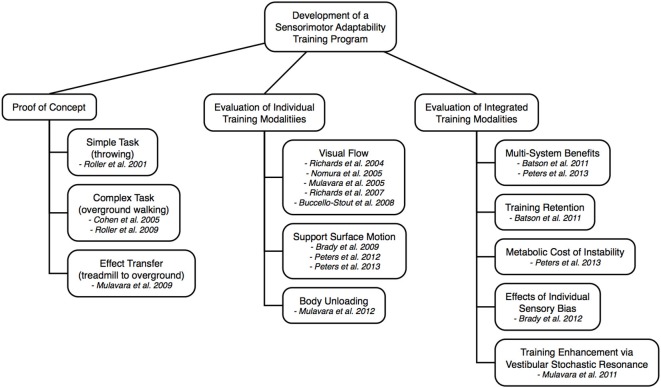
**Schematic overview and corresponding references describing the components and underlying process taken to develop the SA Training Program**.

While developing a SA training program for astronauts we have posed several different questions that are key to determining both the efficacy and application issues that will enable implementation of this countermeasure approach. Figure 2 shows a schematic overview of the central issues that were explored by a series of studies conducted by our laboratory. Each component of the overview is described in the following text.

### Proof of Concept Studies Demonstrating Adaptive Generalization

In an initial proof-of-concept study, we investigated adaptive generalization in a relatively simple throwing task. During a three-week training period, standing subjects threw small balls at a stationary target while wearing lenses that distorted the visual image (Roller et al., 2001). Subjects were randomly assigned to three training groups: (1) wearing undistorted, sham lenses; (2) wearing a single, set of ×2 magnifying lenses; and (3) wearing multiple lenses (×2 magnifying, ×0.5 minifying, and up/down reversing). During the posttest all subjects performed the throwing task while wearing novel, 20° right displacing lenses. Subjects in Group 3, that experienced variable practice training that promoted adaptive generalization, adapted faster to the new visual distortion than those trained with no distortion (sham) or a single distortion (single lens training). They retained their increased adaptability 1 month after completion of the training period. This study confirmed adaptive generalization as a motor learning process that could be applied to enhance motor response adaptability to novel sensory distortions.

Next, we asked if adaptive plasticity of systems controlling a more complex activity, such as obstacle avoidance during locomotion, can also be improved by SA training (Cohen et al., 2005; Roller et al., 2009). In the Cohen et al. (2005) study, subjects wore lenses while they were trained using several challenges involving both walking and standing balance tasks that differed from the criterion obstacle avoidance task. Before and after training they were tested on an obstacle avoidance task that required walking past, stepping over and stepping under obstacles. Time to course completion and the number of touched obstacles were recorded. Subjects who trained with multiple visual distortion lenses (×2 magnifying, 20° right displacing, and up/down reversing lenses) were better able to adapt to novel lenses (×0.5 minifying lenses) than subjects trained with sham lenses or a single visual distortion (20° right displacing lenses). In these studies involving both throwing and walking, subjects who trained with multiple lenses outperformed those who trained repeatedly with a single distortion or with sham lenses. These findings showed that SA training with multiple adaptive challenges increased the ability to learn to learn, facilitating the ability to adapt to a novel sensorimotor environment even for complex tasks like obstacle avoidance during locomotion.

In a follow-up locomotion study, we were interested in determining what critical features of the SA training task are required to achieve adaptive generalization (Mulavara et al., 2009). Normal adults were tested on their ability to walk through a complex obstacle course while wearing novel visual distortion lenses after performing two different training programs comprised of treadmill walking, and standing on a wobble board. They were randomized to three training groups: (1) wearing three different visual distortion lenses (×2.2 magnifying lenses, ×0.5 minifying lenses, and up/down reversing lenses); (2) wearing a single pair of visual distortion lenses (×2.2 magnifying lenses); or (3) wearing undistorted, sham lenses. Following training, all subjects performed the obstacle avoidance task while wearing novel right-shift lenses. Subjects who trained with multiple lenses adapted better to the novel right shift lenses, especially after training on the treadmill. Training for obstacle avoidance during over ground walking in a new sensory environment was successful even under constrained conditions of training on a treadmill that eliminated rotation, linear translation through space, and visual cue salience. Training for dynamic balance with the wobble board alone did not increase training efficacy. For adapting to novel lenses, being exposed to multiple visual distortion lenses was more effective than exposure to only one set or to clear, sham lenses. This finding confirmed the efficacy of using multiple lenses during treadmill training to enhance adaptive generalization in a complex task like over ground walking and obstacle avoidance. In addition, these data showed that treadmill walking is similar enough to over ground walking to serve as its surrogate, and that benefits derived from treadmill training can transfer to more complex ambulatory, over ground tasks. Limitations of training were also demonstrated by the results of the wobble board alone; locomotion is required to achieve the best generalization outcome.

## Evaluation of Individual Training Modalities: Visual Flow, Support Surface Motion and Body Loading

### Variation in Visual Flow as a Training Modality

Multiple sensory inputs, including vision, contribute to locomotor stability. The optic flow field that we observe during self-motion provides cues about our own movement and about the structure of the environment. When a patterned visual scene is presented in a laboratory setting, visual motion and visual polarity are important characteristics that influence how the artificial visual flow is interpreted. The speed of an artificial visual flow pattern has been shown to influence walking speed on a self-driven treadmill (Prokop et al., 1997) and linear flow has been shown to affect postural sway and direction (Bardy et al., 1996, 1999; Warren et al., 1996; Tarr and Warren, 2002). Rotational flow also has an effect. A scene that rolls during quiet standing causes more postural sway when it contains realistic, complex content than when it contains simple patterns (Duh et al., 2002). Over ground walkers immersed in a rolling stereoscopic virtual environment demonstrated compensatory trunk rotation that directed them away from their desired path (Keshner and Kenyon, 2000).

An item is “visually polarized” when it has a distinct axis and its ends are distinctly different (Howard and Childerson, 1994). Most such items, such as buildings, vehicles, and furniture, are vertically asymmetrical with a distinct top and bottom. Humans are accustomed to viewing these objects in a predictable orientation relative to the ground. Visual cues about up-down polarity can effect a viewer’s perceived orientation in much the same way as optic flow. When all items in a field of view are similarly tilted, a viewer who is sitting upright will often feel as if he or she is tilted (Asch and Witkin, 1992). Visual polarity cues can enhance the experience of perceived motion in moving visual scenes. Subjects in a rotating room reported more perceived self-motion when the room contained polarized objects as opposed to when it did not (Howard and Childerson, 1994).

We studied the effects of visual scene rotation and polarity on postural stability while subjects walked on a treadmill (Richards et al., 2004, 2007; Nomura et al., 2005). During treadmill walking subjects viewed a screen onto which virtual visual scenes that yawed, pitched or rolled were projected. Visual scene polarity was achieved by creating a virtual office scene depicting a definitive floor, ceiling and furniture. Reponses to this scene were compared with responses to a scene composed of random dots without any polarization cues. Both polarized and non-polarized rotating visual scenes caused increased variability in trunk motion with visually polarized scenes causing more variability.

Humans are capable of readjusting and recalibrating their gross movements (i.e., walking) so that their estimates to arrive at a target and correct the direction of their movements can be adaptively modified. Rieser and colleagues showed that subjects can accurately estimate the distance to a target and navigate to it without vision. This ability can be re-tuned after exposure to an environment with a new relationship between walking pace and optical flow rate, so humans have an adaptable perceptual motor system with the ability to adjust their strategies to affect desired consequences (Rieser et al., 1995). In a similar study we determined that altered visual flow experienced during treadmill walking produces a lasting after-effect indicating that an adaptive-plastic change has occurred in locomotor function (Mulavara et al., 2005). Therefore, visual flow variation during treadmill walking is an effective way to challenge the locomotor control system and produce an adaptive change in perceptual-motor function. In addition, these data indicated that the amount of visually polarized content is an important factor when designing visual flow patterns to be used for treadmill balance training programs.

Balance control training using variation in visual flow has been used effectively in various clinical populations to improve balance function (Pavlou et al., 2004; Fung et al., 2006; Bugnariu and Fung, 2007; Lamontagne et al., 2007; Buccello-Stout et al., 2008). Pavlou et al. (2004) compared traditional balance training exercises with those consisting of challenges provided by variation in visual flow as a treatment modality for patients with chronic vestibular symptoms. Subjects who received visual flow training had better balance control than subjects given more conventional training. Modification of optic flow increases the complexity of gait variability, which may provide an effective method for rehabilitation (Katsavelis et al., 2010). To determine if SA training using modified visual flow could benefit healthy older adults with postural instabilities associated with age (Buccello-Stout et al., 2008) 16 adults aged 65–85 were randomized into two groups after performing six timed walking trials through an obstacle course on a foam support surface. Then, during eight biweekly, 20 min training sessions of treadmill walking, controls viewed a motionless image on a large screen and experimental subjects viewing a rotating visual scene that simulated repeated travel around the perimeter of a room that induced a sensory conflict that produced gait disturbances. They were post-tested on the obstacle course immediately after training and were retention-tested 4 weeks later. The experimental group had faster times and fewer errors than the control group, and the improvement was retained 4 weeks later. Thus SA training improves balance and gait stability in older adults and can be retained for at least 4 weeks—an encouraging indicator for the general applicability of this method for different populations.

### Variation in Support Surface Motion as a Training Modality

To investigate the potential of using support surface motion variation as a training modality, we had subjects perform treadmill walking on a treadmill mounted on a six degree-of-freedom motion base while viewing a static virtual outdoor scene (Brady et al., 2009). We characterized the individual strategies used by healthy subjects to cope with the support surface perturbations. Strategies fell into two groups: (1) participants who fixed themselves relative to space (FIS); and (2) participants who fixed themselves relative to the support base (FTB). FIS subjects allowed the treadmill belt to move laterally beneath them but did not center themselves on the belt as it moved. FTB subjects moved with the treadmill, keeping themselves centered on the belt as the treadmill moved from side to side. The degree of fixation varied across subjects in both groups. So, normal adults have individual preferences for optimizing stability, either relying more heavily on vision or relying more on non-visual sensory input. The adaptive responses in gait control could be produced with relatively short exposures (20–30 min.) to the unstable walking surface similar to the time frame that produced adaptive responses after locomotion while viewing a rotating visual scene described above supporting the use of an unstable surface as a training modality.

To determine if some people are more naturally prone to find successful gait adaptation techniques than others, we measured stride frequency in subjects who walked on our oscillating treadmill system (Peters et al., 2012). To investigate individual adaptive gait responses, we tested subjects’ responses to lateral oscillation of the treadmill over 20 min, of walking at 1.1 m/s. Twenty-five percent of participants showed a consistent, entrained strategy. Subjects who did not entrain used several alternate techniques to adapt to the novel locomotor environment. The unique and varied locomotor responses we observed reinforces the concept that multiple solutions can be used to solve a single adaptive problem. Training may facilitate selection of appropriate solutions matched to each subject’s unique sensory biases and adaptive capabilities.

### Variation in Body Loading as a Training Modality

Body-load sensing plays a central role in the control of gait and postural equilibrium. The central nervous system (CNS) receives afferent input from Golgi tendon organs, muscle spindles in the ankle, knee, and hip, Ruffini endings, and Pacinian corpuscles in the soles of the feet, and integrates this information with visual and vestibular input to control locomotion. For example, during walking, when a limb is loaded, receptors that are activated include those from the foot, muscles, joints, and the Golgi tendon organs of the extensors, which are the primary load receptors. Body load sensing is also important for controlling balance and posture during locomotion, by shaping motor output patterns during stepping and the termination of locomotion (Dietz, 1996; Harkema et al., 1997; Layne et al., 1998; Dietz et al., 2002).

To determine if variation in body loading could be used as a training modality we investigated how the locomotor control system responds after a period of exposure to body unloading during treadmill walking (Ruttley, 2007; Mulavara et al., 2012). After only 30 min of exposure to 40% body weight support subjects showed adaptive alteration in various gait parameters during treadmill walking immediately following the period of body unloading. Thus, similar to variations in visual flow and support surface motion, a short period of treadmill training can elicit an adaptive response in the body’s load sensing systems that control locomotion and therefore can be used as an SA training modality.

## Evaluation of Integrated Training Modalities

### Multisystem Benefits and Training Retention

The next series of studies evaluated the integration of the training modalities described above using both alterations in visual flow and support surface motion in combination to produce SA training. In an initial study we aimed to determine if SA training benefits derived from exposure to modified visual and support surface motion could transfer to a new discordant sensory experience and how long any learning benefits would be retained (Batson et al., 2011). The training group completed three, 31 min training sessions that included congruent and incongruent visual flow along with support surface movement during treadmill walking on the six degree-of-freedom motion base with 5 min exposures to various combinations of visual and support surface perturbations. The control group walked on the treadmill but experienced no visual alterations or support surface perturbations, although their exposure time was broken into blocks to mirror the condition used for the training group. All subjects were post-tested on a novel Transfer/Retention profile at 20 min, 1 week, and 1, 3 and 6 months after their final training sessions. Stride frequency was used to assess locomotor stability and an auditory reaction time task characterized the cognitive resources required to maintain balance. When compared to controls SA-trained subjects had enhanced locomotor stability and reduced cognitive cost of adaptation. Interestingly, trained subjects maintained their level of performance when tested 6 months later. We attribute this result to the trained subjects’ ability to apply the adaptive techniques they developed during their training sessions.

In summary, results indicated that SA training, using a combination of modified visual flow and support surface motion, enhanced the ability of subjects to rapidly adapt locomotor function to allow stable walking in a novel, discordant sensory environment at a lower cognitive cost. This improved performance could be retained over a 6 month period and perhaps longer, indicating that a component of this training could take place before long-duration space missions. Bhatt and Pai (2009) reported similar findings in terms of retention of training when they investigated repeated-slip training. Participants who were slipped multiple times showed improvements in balance and stability metrics at a single-slip test session performed 4 months after their first. Another group that received only one slip during the first session, followed by another single slip 1 week, 2 weeks, and 1 month later, also improved at the 4 month follow-up. The retention performance of both groups was comparable. In addition, rapid relearning of the slip recovery skill was also demonstrated even after a 12 month duration between training and testing (Bhatt and Pai, 2005). These studies indicate that even short, repeated training exposures can produce skills that are retained long-term.

### Training and the Metabolic Costs of Instability

Humans naturally select gait patterns that minimize their energetic costs (Cavanagh and Williams, 1982; Cavanagh and Kram, 1985). Ortega and Farley (2007) determined that, during walking, the elderly consume more metabolic energy than the young and that the difference between groups is not attributable to a disparity in limb work. They suggested that differences in the metabolic cost of stabilizing the body contribute to the loss of efficiency. Similarly, we believe that when discordant sensory input is destabilizing enough to disrupt gait, any strategy used to maintain balance while walking will be associated with elevated energetic costs, perhaps because of the increased muscular co-contraction required to deal with instability (Frost et al., 2002). Finley et al. (2013) showed that locomotor adaptation to split-belt treadmill walking was associated with a reduction in the metabolic power associated with walking. These adaptive responses included a reduction in step length asymmetry accompanied by a simultaneous, bilateral reduction in lower limb muscle activity. These data support the concept that the CNS is able to rapidly optimize walking patterns to reduce energy cost during exposure to novel and dynamic walking challenges.

We studied changes in metabolic cost associated with a locomotor adaptation training paradigm. To capture the broad effects of destabilized walking and the subsequent adaptive response, we collected metabolic, stride frequency and reaction time data. Shorter, faster steps are an indicator of locomotor instability (Batson et al., 2011), so we used stride frequency to quantify this measure. We quantified the additional cognitive resources required to maintain postural stability during support-surface perturbation with an auditory reaction time task. Metabolic cost was measured with a portable gas analysis system. Our subjects performed an 8 min, 4.0 km/h walk on our treadmill/motion base system while receiving no perturbations (baseline condition), and then they completed a 20 min walk at the same speed while the motion base oscillated mediolaterally at 0.3 Hz, ±25.4 cm. At the beginning of the perturbation period, stride frequency, auditory reaction time, and VO_2_ increased, indicating increased balance disruption, cognitive load, and metabolic cost. All parameters gradually decreased as the subjects adapted during the 20 min period.

The observed decrements are operationally meaningful in our application because they illuminate broader implications for the postflight locomotor instability that is commonly observed in returning astronauts. Until recently, locomotor adaptation to discordant sensory conditions has been characterized primarily in terms of impact on the underlying mechanisms contributing to locomotor stability. These results indicate that uncoordinated walking during periods of adaptive change in these conditions also comes at significant cognitive and metabolic costs to the crew. Cognitive load increases and metabolic cost rises because of new demands on attention and additional physical work required to maintain balance while walking. We are encouraged that SA training can impart performance benefits to the parameters required to successfully execute mission critical activities. Energetic cost is a key contributor to the duration and intensity of extravehicular activities (EVAs) performed by suited astronauts, and previous research on suited locomotion has explored the effects of load, slope, and walking vs. running (Carr and Newman, 2007a,b). Therefore, a successful training countermeasure will not only impart gains in locomotor stability but may also increase productivity during missions by extending work time in extravehicular activity suits by lowering the metabolic cost experienced during locomotor adaptation to new gravity environments.

### Effects of Individual Sensory Bias

Healthy adults integrate visual, vestibular, and somatosensory information to produce appropriate motor output. The degree to which these sensory inputs are weighted and reorganized in discordant conditions varies by individual. Sensory weighting preferences have been reported for special populations and observed in healthy individuals under certain conditions. For example, higher visual dependence has been documented for stroke patients (Bonan et al., 2004; de Haart et al., 2004), astronauts (Young and Shelhamer, 1990) elderly people (van Hedel and Dietz, 2004), and vestibularly-intact but anxious normals (Viaud-Delmon et al., 2000). Vestibular weighting has been shown to increase just before initiating a turn while walking (Kennedy et al., 2005) and may be more prevalent in individuals who suffer chronic headaches (So and Bent, 2009). Autistic children (Masterton and Biederman, 1983) and individuals susceptible to mal de debarquement symptoms (Nachum et al., 2004) depend more heavily on somatosensory cues. Approximately 30% of healthy normals are “highly” visually dependent (Warren et al., 1996; Keshner et al., 2004; Streepey et al., 2007; Brady et al., 2009).

We have previously shown that healthy adults walking in novel discordant conditions display inherent differences in how they weigh visual information (Brady et al., 2009). We have explored whether a person’s walking performance in a new discordant sensory environment could be predicted based on his or her inherent level of visual dependency (Brady et al., 2012). To characterize an individual’s level of visual dependency, we measured trunk translation via a motion capture system while subjects walked on a treadmill for 5 min (1.1 m/s) and traversed a “virtual” hallway that oscillated laterally on a large screen in front of them. Following the 5 min visual dependency test, subjects performed training across three sessions that included congruent and incongruent visual flow along with sinusoidal lateral support surface movement during treadmill walking as before. Following the training, subjects walked with novel sensorimotor stimuli consisting of a forward visual flow rate that was doubled and a sinusoidal roll of the support surface and visual scene that were 90° out of phase with each other. The subjects who demonstrated greater visual dependency had increased stride frequency and reaction times when exposed to the novel sensory discordant condition, indicating that these subjects had decreased postural stability and increased cognitive load when negotiating novel discordant conditions. These data indicate that subjects who are more reliant on vision for control of movement have more difficulty adapting their walking strategies in new environments and this was manifested across a number of performance modalities. A high level of visual dependency might therefore predict a decreased ability to adapt to novel environments. Other individual sensory biases including vestibular and proprioceptive bias may also predict SA but details of those biases remain unclear. All motor responses exhibit some degree of variability during multiple repetitions of a given task and have been previously thought to be a random process. Recent studies, however, suggest that such variability may represent a deliberate, actively regulated process that can facilitate motor adaptability (Davids et al., 2003; Stergiou and Decker, 2011; Herzfeld and Shadmehr, 2014; Wu et al., 2014). Indeed, baseline inter-trial correlations and adaptability in the saccadic oculomotor system are strongly related (Wong and Shelhamer, 2014) suggesting that inherent individual variability may predict SA and could be another tool to fine tune training countermeasures approaches.

Astronauts show significant inter-subject variations in their abilities to adapt to microgravity and to readapt to Earth’s gravitational environment. An open question persists as to what contributes to this individual variability in adaptive capability. Can we predict, preflight, individuals who will have greater difficulty adapting to gravitational transitions? More importantly, can we use information regarding individual differences in adaptive ability to design and implement customized countermeasures to facilitate adaptation? Developing predictive measures of SA would allow us to optimize training prescriptions by designing and implementing SA training countermeasures that would be customized for each astronaut’s unique sensory bias and individual adaptive capabilities. Customization would allow more efficient use of crew time during training and produce better outcomes.

### Vestibular Stochastic Resonance: a Potential Enhancer of SA Training

Stochastic resonance (SR) occurs when a non-linear system exhibits a stronger response to a weak input when there is a certain, non-zero level of noise present (Collins et al., 1995, 2003; Moss et al., 2004; McDonnell and Abbott, 2009; Aihara et al., 2010). Information flow is enhanced with the inclusion of this non-zero noise level (Collins et al., 2003). Applied to the soles of the feet as sub or peri-threshold mechanical vibration, SR noise improves postural stability (Priplata et al., 2002, 2006). SR as electrical noise applied to the knee (Gravelle et al., 2002) or to the paraspinal muscles (Reeves et al., 2009) is also effective. Imperceptible stochastic input applied in this manner to the vestibular system (Stochastic Vestibular Stimulation, SVS) of normal subjects improved the association between the imposed weak central venous pressure oscillations and heart rate responses (Soma et al., 2003). Parkinson’s patients demonstrated a 4.5% improvement in balance function with SVS applied at 0.1 mA (Pal et al., 2009), and we saw balance improvements in the 5–26% range in healthy normals, standing without vision on a compliant surface, when SVS was applied between 100–400 microamps (Mulavara et al., 2011). Presumably, these gains occur because of enhanced signal detection by the vestibular system. Some studies have shown significant improvement in postural balance control aiding recovery when electrical or mechanical SR stimulation was given to the muscles across the ankle joints in conjunction with conventional coordination training compared to training alone (Ross and Guskiewicz, 2006; Ross et al., 2007, 2013; Ross, 2007).

In the present context, SVS might be used as an adjunct to SA training. One example would be to improve the adaptive performance of visually dependent subjects. Visually dependent subjects exhibit less capacity for adaptation and might benefit from personalized training to reduce their visual dependency and increase their reliance on vestibular inputs. The training program might include two components: (1) walking on a treadmill/motion-base system and watching discordant visual scenery to reduce dependency on vision along with support-surface motion to challenge gait stability; and (2) the same training supplemented with SVS to enhance vestibular signal detection. These two components should act in synergy during training to reduce visual dependency while enhancing the use of vestibular information. We speculate that an individualized training program designed to decrease dependency on a single sensory source and to promote use of multiple sensory modalities will enhance the individual’s ability to adapt to a novel discordant sensory environment.

## Conclusions and Countermeasure Recommendations

The following points summarize the research conclusions and provide recommendations that inform the development of treadmill-based SA training systems:

• SA training with multiple challenges increases adaptive generalization, facilitating the ability to adapt to a novel sensorimotor environment not previously experienced even for complex tasks like obstacle avoidance during over ground locomotion.
• Altered visual flow, body loading and variation in support surface motion experienced during treadmill training produces adaptive changes in locomotor function over relatively short periods of exposure (20–30 min.) and therefore can be used as effective SA training modalities.
• SA training improves locomotor adaptability; increasing stability, lowering cognitive cost and reducing the metabolic expenditure during adaptation to novel discordant sensory conditions.
• SA training on a treadmill is similar enough to over ground walking to be an effective training modality that transfers to more complex over ground ambulatory tasks (i.e., obstacle avoidance).
• Visual dependency is a predictor for decreased SA. Developing predictive measures of SA would allow us to optimize training prescriptions by designing and implementing SA training countermeasures that would be customized for each astronaut’s unique sensory biases and individual adaptive capabilities.
• SVS, could be used as a means to augment adaptive responses during SA training improving the efficacy of training.

These recommendations will be useful in the design of any countermeasure system or regimen used to prepare for exploration-class space missions. We envision that the final countermeasure will use a virtual reality system coupled with multi-direction treadmill that will allow the user to walk in any direction in a varied and interesting virtual environment. This type of fusion interface, which incorporates both virtual and non-virtual devices across sensory modalities, produces multi-sensory, virtually augmented, synthetic environments. These synthetic environments can serve as pre- and inflight training tools providing sufficient sensorimotor challenge to astronauts and to maximize their motor response adaptability in preparation for various gravitational transitions. Given inflight constraints on time allocated for exercise during space missions we propose that SA training could begin before missions during the preflight training period. Therefore one can conceive of this training more in terms of a preflight “inoculation” that may only require infrequent “booster” training to maintain increased adaptability. Finally, a collateral benefit of the application of SA training, will be to make training programs more interesting, which lends itself to participant compliance and enhanced psycho-social benefits (Annesi and Mazas, 1997).

## Conflict of Interest Statement

The authors declare that the research was conducted in the absence of any commercial or financial relationships that could be construed as a potential conflict of interest.
